# Paired-pulse tactile stimulation enhances tactile perception in neurotypical but not autistic children

**DOI:** 10.21203/rs.3.rs-11032804/v1

**Published:** 2026-10-06

**Authors:** Matea Balabanovska, Jason L. He, Helen J. Powell, Richard A. E. Edden, Stewart H. Mostofsky, Nicolaas A. Puts

**Affiliations:** 1. Department of Forensic and Neurodevelopmental Sciences, Institute of Psychiatry, Psychology, and Neuroscience, King’s College London, London, United Kingdom; 2. Russell H. Morgan Department. of Radiology and Radiological Science, Johns Hopkins University School of Medicine, Baltimore, USA; 3. F.M. Kirby Center for Functional Neuroimaging, Kennedy Krieger Institute, Baltimore, USA; 4. Center for Neurodevelopmental and Imaging Research, Kennedy Krieger Institute; 5. Department Of Psychiatry and Behavioural Sciences, Johns Hopkins University School of Medicine, Baltimore, USA; 6. MRC Centre for Neurodevelopmental Disorders, King’s College London, London, United Kingdom

**Keywords:** Autism, tactile perception, sensory processing, psychophysics

## Abstract

Tactile sensory differences are common in autism. Sensory gating, a mechanism by which perception is filtered, is of relevance, yet it is unknown if atypical sensory filtering in autistic people directly impacts the perception of sensory stimuli. In this study we used a novel psychophysical protocol to examine the effect of pre-pulse sub-threshold stimulation on tactile detection thresholds in 8–12 year old autistic (n = 24) and neurotypical (n = 51) children. We used two pre-pulse conditions with either a 30 ms or 100 ms interval. We hypothesised, based on prior work, that sub-threshold stimulation would worsen detection threshold in neurotypical controls whereas the effect of sub-threshold stimulation would be absent in autism. We further hypothesised that the absence of gating would correlate with autism outcomes. Contrary to these expectations, we observed that both pre-pulse stimulation conditions significantly improved detection thresholds in neurotypical children but not autistic children. The improvement is consistent with recent work examining the impact of different pulse types and intervals and reflects the importance of sensory filtering mechanisms on tactile perception. These findings reinforce prior findings that sensory filtering may underlie poorer perceptual capacity in autism. We did not find associations with clinical measures suggesting that the relationship between perceptual and behavioural sensory differences and their relation to autistic traits is complex. This work shows that atypical sensory gating is directly related to differences in perception in autistic children. This work has strong implications for understanding how different brain mechanisms give rise to altered sensory processing, core to autism.

## INTRODUCTION

Autism Spectrum Condition (ASC) henceforth autism is characterized by social-communication difficulties, repetitive, restricted behaviours (RRB) and sensory differences. Recent reports suggest that autism has a prevalence between 2–3% and that 40–95% of autistic people experience functional impact due to sensory differences (Lane, 2020). Tactile sensory differences are among the most prevalent and can impose challenges with basic tasks such as dressing up or sleeping(Lane, 2020; Mikkelsen et al., 2018; Puts & Cascio, 2023). The critical role of touch during development is well-established and tactile differences, which range from perceptual to behavioural level, can have a direct impact on social interactions (He et al., 2021; Lane, 2020).

While sensory differences are commonly assessed through questionnaires, these approaches conflate differences in perception and emotional reactivity, making it difficult to establish the nature of sensory differences(He et al., 2023). One way to capture tactile differences is through psychophysics. Psychophysics is the study of the relationship between stimulus and behaviour and allows for objective testing of an individual’s sensitivity to specific stimulus parameters, including detection thresholds, tactile discrimination, accuracy, and adaptation (Blakemore et al., 2006; Cascio et al., 2008; Puts et al., 2014; Tannan et al., 2008; Tavassoli et al., 2016). These metrics offer measures of tactile perception which are directly translatable to biological mechanisms underlying encoding of the physical parameters of interest. For example, a two-point discrimination task is known to rely on lateral inhibition(Tommerdahl et al., 2010).

Atypical tactile processing in autism has been reported on a variety of these metrics including static detection and tactile discrimination (Cascio et al., 2008; He et al., 2021; Robertson & Baron-Cohen, 2017; Tavassoli et al., 2016).

One understudied aspect of tactile perception is that of habituation, or more specifically, “adaptation”. Habituation, or ‘adaptation’ is a mechanism by which the response to a stimulus is modulated by prior exposure (Kohn, 2007; Kohn & Whitsel, 2002; Tommerdahl et al., 2010). In some instances, the response diminishes, whereas in others, the brain preferentially responds to the repeated stimulus properties (Goble & Hollins, 1994). Sporadic reports on failure to adapt to repeated stimulation in autism indicate that perceptual atypicalites go beyond atypical sensory processing to single stimulation and point towards altered mechanisms of adaptation to changes in the sensory environment (S. A. Green et al., 2015; McKernan et al., 2021; Puts et al., 2014; Tommerdahl et al., 2010).

Of particular interest here is sensory gating, which relates to the filtering and subsequent perception of incoming sensory stimuli; i.e., whether we are able to detect touch or not. Although altered sensory gating has been associated with autism (Khan et al., 2016; Piccardi et al., 2021), few studies have examined the effect of altered sensory gating on tactile perception. Previous research has shown that detection threshold is increased in neurotypical children when they are asked to respond when they perceive a stimulus that starts subliminally and is increased in amplitude (Puts et al., 2013). Detection thresholds were worse in autistic children and they did not show modulation of threshold (Puts et al., 2017, 2014). However, this task is somewhat biased due to a strong attentional and reaction-time components, since children were asked to respond upon perception of a stimulus. An alternative way to examine sub-threshold adaptation is through more conventional paired-pulse tasks that do not contain a reaction time component. However, these paired-pulse, or pre-pulse, tasks are typically done passively and electrophysiologically, rather than measuring their impact on behaviour.

Two previous studies have reported the impact of subliminal stimulation on tactile perception in neurotypical adults and showed that a subliminal (i.e. not consciously perceptible) tactile pre-pulse changed the detectability of a subsequent tactile test pulse (Blankenburg et al., 2003; Iliopoulos et al., 2020). One study included a paradigm that accounted for single or repeated (1s pulse-train) administration of the pre-pulse prior to the test stimulus (Iliopoulos et al., 2020). Repeated stimulation consistently worsened detection of the test pulse whereas, detection of the test pulse was not affected, or even enhanced, when the test pulse was preceded by single subliminal pulse. These findings show that different mechanisms can be targeted by modulating the pre-pulse parameters as single and repeated administration of pre-pulses evokes different perceptual effects (Iliopoulos et al., 2020) and may reflect different aspects of cortical inhibition. Indeed, pre-pulse inhibition work in both the animal and human domain has reported that the effect of the interstimulus interval between pre-and test pulse engages different GABAergic processes (Brickwedde et al., 2019; Connors et al., 1988; Schmidt-Wilcke et al., 2018). In the context of autism, this is relevant to the theory of altered excitation/inhibition balance (Coghlan et al., 2012; J. Green & Garg, 2018; LeBlanc & Fagiolini, 2011; Rubenstein & Merzenich, 2003). To date, there have been few investigations of the effect of pre-stimulation on the ability to detect tactile stimulation in autism. Given the literature of inhibition imbalance in autism, the role of inhibition in driving adaptation and sensory gating, and altered sensory sensitivity in autism, it is possible that the lack of inhibition found in autism is associated with lack of gating of sensory detection. Examining the differential impact of stimulation parameters of adaptation may have strong impact of understanding the biology underlying altered tactile perception in autism.

Based on prior work by us and others, autistic children may have less restricted sensory gating mechanisms leading to difficulties in sensory perception, with potential clinical impact. Establishing altered adaptation at the level of perception of touch may allow for differentiating autism and inform potential targeted/disorder-specific interventions. By modulating specific stimulus parameters (the type of stimulus; duration of adaptation (30 or 100 ms based on prior work) we can gain insights into the underlying biology of altered touch perception in autism which may inform our understanding of the heterogeneity of autism and sensory differences.

Here we aim to 1) Investigate group differences in the extent of pre-pulse inhibition on detection threshold in 8–12 year-old autistic children and neurotypical controls. 2) Investigate the effect of interstimulus interval between pre-pulse and test pulse on detection threshold to determine specific mechanisms underlying low-level adaptation. 3) To investigate whether measures of detection threshold are related to clinical manifestations of core sensory and social domains of autism.

Based on prior paired-pulse work and on previous findings showing poorer performance on a dynamic detection task compared to detection thresholds measured without subliminal stimulation, we hypothesize that adaptation through pre-pulse stimulation leads to increased (worse) detection threshold in neurotypical children, and thus, that the detection threshold would be higher in pre-pulse conditions. Based on prior work showing reduced or absent adaptation and sensory gating in autism we expect detection thresholds not to change compared to the unadapted condition. We hypothesise a lack of pre-pulse inhibition to be associated with sensory differences as captured by the SEQ and core autism outcomes captured by the Autism Diagnostic Observation Schedule (ADOS-2) with those with reduced adaptation showing more severe symptoms.

## METHODS

### 2.1. Participants

Twenty-four children with a diagnosis of autism and fifty-one children without a diagnosis of autism or other conditions (here referred to as neurotypical), took part in the study. Informed consent was provided by caregivers on behalf of the children. Data were drawn from studies under the ethical approval of the Kennedy Krieger Institute and the Johns Hopkins School of Medicine Institutional Review Boards.

The recruitment strategy and inclusion/exclusion criteria can be found in Supplement S1.1. and have already been described at length elsewhere (He et al., 2021). Autism diagnosis was based on assessments using the Wechsler Intelligence Scale for Children (WISC-5) (Wechsler, 2014), ADOS-2 (Lord et al., 2000), and then confirmed through clinical assessment. Participants in the control group were determined to not have psychopathology using the Diagnostic Interview for Children and Adolescents Fourth Edition (DICA-IV) (Reich, 2000) or/and the Kiddie Schedule for Affective Disorders and Schizophrenia (Kaufman & Schweder, 2004). Relevant descriptive statistics for participants in each group are presented in Table 1.

### 2.2. Tasks

#### 2.2.1. Psychophysical assessment of vibrotactile perception.

All stimulation was presented using a Cortical Metrics tactile stimulator (CM4) delivered to the left index and middle finger using two alternative forced choice paradigms (Puts et al., 2013; He et al., 2021). Participants completed detection protocols consisting of static detection without pre-pulse (SDT) and static detection with pre-pulses delivered with either a 30 ms (SDT30) or 100 ms (SDT100) interstimulus interval (ISI) between pre-pulse and test-stimulus. A visual schematic of these protocols is presented in Figure 1.

##### Static detection.

In the static detection protocol, a vibrotactile stimulus was pseudorandomly administered to either the left index or middle finger (frequency = 25 Hz; starting amplitude = 20 μm; duration = 500 ms, 24 trials). In each trial the participants reported on which finger they felt the stimulation. Prior the task, participants did three consecutive practise trials that necessitated accurate responses to ensure that the protocol was understood. The final static detection threshold was determined using the average stimulus amplitude of the last five trials (Holden et al., 2012; He et al., 2021)

##### Static detection with pre-pulses.

In the pre-pulse detection protocol an imperceptible pre-pulse stimulus (frequency = 25 Hz; amplitude = 3 μm; duration = 40 ms) preceded the static detection stimulus (frequency = 25 Hz; starting amplitude = 20 ms; duration = 500 ms, 24 trials). The amplitude was chosen based on pilot work showing 3 μm to be beyond the perceptible amplitude for all participants. There were two conditions of the pre-pulse protocol based on the latency between the pre-pulse and the main pulse. In the first condition the pre-pulse was administered 30 ms before the main pulse (SDT30). In the second condition the pre-pulse was administered 100 ms before the main pulse (SDT100). Besides the addition of the pre-pulse, the protocol was identical to the static detection protocol (SDT) (Fig. 1). Conditions were presented separately, sequentially, and counterbalanced.

#### 2.2.2 Clinical assessments

**Autism Diagnostic Observation Schedule (ADOS-2)** is a standard autism diagnostic symptom measure with good external validity. The assessment includes a researcher-child interaction during which the researcher uses a standardized set of social tasks. The current study used ADOS-2 Modules 3 and 4 because the cohort was verbally fluent.

**The Sensory Experiences Questionnaire (SEQ)** is parent-rated questionnaire which aims to capture broad range of sensory differences that an individual experiences in daily life. The SEQ has a total of 105 questions pertaining to sensory experiences which are scored on a 5-point Likert scale ranging from “Never” to “Always”. The questionnaire has three core domains; hyperreactivity, hyporeactivity and sensory seeking.

**The Social Responsiveness Scale (SRS)** is a questionnaire that measures autistic traits. The SRS has 65 items that are scored on a 3-point scale ranging from 0 (never true) to 3 (almost always true).

**The Repetitive Behaviours Scale – Revised (RBS-R)** is a questionnaire that measures the level of repetitive behaviours in autistic children and adults. The RBS-R has 44 items and behaviours are rated on a 4-point scale ranging from 0 (behaviour does not occur) to 3(behaviour occurs and is a severe problem).

### 2.3 Study design

Clinical assessment and psychophysical tasks were administered on separate days, but within one month of each other.

Participants performed the tasks detailed above in a dedicated and comfortable testing room. They were asked to place all fingers of their left hand on each of the sensors but only the left index and middle finger received tactile stimuli. Participants responded to each protocol using the corresponding fingers of the right-hand fingers 2 and 3. All stimulation was assessed on the non-dominant hand as is common in tactile psychophysics (as between-participant variance is thought to be higher in the dominant hand). As this work was part of a larger study that also assessed brain levels of GABA and Glutamate in the sensorimotor cortex, stimulation location was kept constant to allow for consistent MRI voxel placement. Handedness was measured using the Edinburgh handedness inventory. Participants could request as many breaks as needed between the protocols.

### 2.4. Analysis

Case-control data were analysed in R. All data were normally distributed. Participants with poor tracking (poor convergence) were excluded (one NT for SDT, one for SDT30, and one for SDT100). First, we compared groups on metrics including age, IQ captured by WISC total and sub-domain scores, and sensory differences captured by SEQ scores using a Student T-test with alpha set to 0.05. Detection threshold was our primary outcome variable. For our primary hypotheses, to assess whether there was an effect of interstimulus interval and of diagnosis, we used a repeated measures ANOVA using the *aov* function and Tukey’s LSD with threshold as dependent variable, and diagnosis (autism or neurotypical) and condition (repeated factor, SDT, SDT30, and SDT100) as independent variables. We additionally added a diagnosis by condition interaction. The *effectsize* package was used to determine partial η^2^. As per literature recommendation, IQ was not entered as a covariate (Dennis et al., 2009; and 4.4.). Pearson correlation analyses were used to determine the relationship between each of the three different conditions (SDT, SDT30, and SDT100) as well as the ‘sensory gating’ change scores (percentage difference between SDT and SDT30, and percentage difference between SDT30 and SDT100) and our secondary outcome variables, ADOS-2 and SEQ (hyperresponsivity, hyporesponsivity, sensory seeking) scores to assess the relationship between perception and clinical symptoms. We first performed these associations across the cohort and then for each group independently when possible. Given that ADOS scores are associated, SEQ sub-scores are associated, and thresholds are associated, and this is an exploratory analysis, alpha was set to 0.05. R was used for visualisation using raincloud approaches (Allen et al., 2021).

## 3. RESULTS

Independent T-test analysis showed that there were no significant differences between the autism and neurotypical groups in age. Given the small age range, age was not further considered in our analysis. IQ, as captured by the WISC-5 scale, was significantly different in full scale IQ scores (t = 2.037, df = 33.996, p = 0.050), processing speed index (t = 2.194, df = 37.846, p = 0.034), verbal comprehension index (t = 2.104, df = 35.108, p = 0.043). There were no significant differences between autism and neurotypical groups in the fluid reasoning index, visuospatial index and the working memory index of the WISC-5 (Table 1).

Significant differences between groups were also found in the total subdomain scores for the SEQ: total hyperreactivity (t = 9.455, df = 24.760, p < 0.001), total hyporeactivity (t = 2.227, df = 68, p = 0.029) and total sensory seeking (t = 5.803, df = 27.020, p < 0.001) (Table 1).

### 3.1 Analysis of psychophysical assessment

The results from the pre-pulse static detection protocol are illustrated in Figure 2. A 2×3 repeated measures ANOVA with diagnosis (autism, and neurotypical) and condition (SDT, SDT30, SDT100) as independent factors and threshold as dependent variable showed no significant interaction between group and diagnosis (F = 1.105, p = 0.33, partial η^2^ < 0.01). We then considered the main effects in the context of a non-significant interaction and found a significant main effect of condition (F = 8.05 p < 0.001) with medium effect size partial η^2^ = 0.07), and a significant main effect of diagnosis (F = 9.53, p < 0.002) with small effect size (partial η^2^ = 0.04). These data suggest that both groups showed a similar effect of condition and thus, of pre-pulse threshold. Post-hoc testing using Tukey’s LSD confirmed that the autism group had higher thresholds (p = 0.002; consistent with our prior work) than neurotypicals. When observed across groups together, we observe a decrease in detection threshold SDT30 and SDT100 conditions (SDT30, p < 0.0001; SDT100, p < 0.01) but not between paired-pulse conditions (p = 0.24). Qualitative assessment (Figure 2B–E) shows the reduction in detection thresholds in the pre-pulse conditions appears more consistent in the neurotypical group. Indeed, when examined per diagnostic group independently, only neurotypicals showed a significant effect of condition (F = 7.82, p < 0.001) but not the autistic group (F = 1.99, p = 0.14). Post-hoc T-tests are reported in Figure 2B–E showing significant changes in neurotypicals in both conditions, but this is only moderately significant for SDT30 in the autistic group, but not for SDT100, and more variable.

We then also compared these findings to the dynamic detection threshold data for a subset of participants, and found, using a simple repeated measures ANOVA within-group with Tukey LSD posthoc testing, that dynamic detection threshold was higher than baseline SDT for non-autistic children (p > 0.001), but not for autistic children (p = 0.12) as previously reported. In autistic children, SDT30 (p = 0.001) and SDT100 (p = 0.041) thresholds were reduced compared to DDT. SDT, SDT30 and SDT100 thresholds were lower than DDT in neurotypical children (all p < 0.001). See Supplementarily Figure 3.1.

Given that we hypothesised that 30 and 100 ms ISI reflect different neuronal mechanisms, we explored whether the change in performance in the SDT30 task was associated with performance in the SDT100 task. We observed that performance in the 30 ms condition and 100 ms condition were significantly associated with baseline performance in neurotypicals, but not significantly in the autism group (Figure 3).

We then explored if there was anything phenotypically distinct in those who showed no change or worse thresholds in either SDT30 or SDT100, or both, but no differences were found in SEQ or ADOS-2 scores (Supplement S2, Table S2).

### 3.2 Analysis between clinical and psychophysical assessments

We did not find any significant associations between thresholds or change scores and clinical symptoms on the ADOS-2 or SEQ subdomains (Figure 4), none of these correlations were significant at alpha = 0.05). Prior work (He et al., 2021) showed a significant across-cohort correlation between SDT and SEQ hyper- and hyporeactivity in a larger sample. In Figure 4 we show that we were not able to reproduce these findings in this smaller cohort. The only significant finding was a negative association between absolute change in the SDT30 condition compared to baseline, and hyporeactivity, whereby neurotypicals with lower hyporeactivity showed a smaller absolute change, but this would not pass correction for multiple comparisons (R = −0.39, p = 0.021).

## DISCUSSION

In this study we tested the impact of pre-pulse inhibition directly on sensory perception as measured through detection threshold in autistic and neurotypical children. We used a novel pre-pulse detection threshold protocol to examine specific mechanisms underlying low-level adaptation. We show that detection thresholds in neurotypicals are affected by different pre-pulse inhibition parameters, whereas these did not significantly affect autistic children who in comparison, had elevated detection thresholds. We found that differences in tactile perception are potentially due to atypical sensory gating mechanisms, with immediate impact on understanding sensory differences in autism. However, we did not find any significant associations between detection thresholds from each condition with sensory differences captured by the SEQ and autism traits captured by ADOS-2, RBR, and SRS.

### Pre-pulse stimulation enhances detection threshold in neurotypical but not autistic children

The finding of improved detection threshold after sub-threshold stimulation in neurotypical children was surprising. We initially hypothesized thresholds to increase (get worse) after pre-pulse adaptation. This hypothesis was based on prior work in which children were asked to indicate when they perceived a stimulus dynamically increased in amplitude, which led to worse detection threshold compared to baseline static detection threshold as performed here (He et al., 2021; Puts et al., 2014). The underlying theory was that subthreshold stimulation towards threshold induces inhibitory interneurons which supress the neural activity required for perception (Favorov & Kursun, 2011; Zhang et al., 2011), yet without the reaction time bias in the dynamic task. The hypothesized effect of the sub-threshold pulse was further supported by TMS paired-pulse work showing that a subthreshold pulse decreases the size of motor evoked potentials (Gilbert, 2017; Vlachos et al., 2017). It was anticipated that the task in this study (SDT30 and 100) would do the same.

Contrary to our hypotheses however, thresholds were significantly reduced compared to baseline in both pre-pulse conditions, which is in line with two other healthy human adult studies (Blankenburg et al., 2003; Iliopoulos et al., 2020) showing the enhancing impact of a single imperceptible stimulus on threshold. Our work shows that these effects were only present in typically developing children, but not in autistic children. It may be the case that the worsening of threshold in neurotypicals the dynamic task showed in a previous study (He et al., 2021; Puts et al., 2013) was more akin to the repeated prestimulus 1s trains as described in Iliopoulos et al. (2020) that also led to worsening of threshold and indeed, in neurotypical children, DDT significantly worsened thresholds in neurotypicals but, as shown before (Puts et al. 2014), not in autistic children. Consistent with prior work, our data showed worse baseline detection threshold in autistic children when compared to neurotypical groups in larger samples (He et al., 2021; Puts et al., 2014; Tavassoli et al., 2016).

While there are clear main effects of condition and detection thresholds in both groups it is important to note that certain individuals do get worse as observed on the individual plots. Comparison of characteristics showed no differences between these individuals and characteristics of the overall cohort. Considering that 64% (18/28) of the individuals were from the neurotypical group, it is possible that these individual deviations in the static detection thresholds reflect a bottom effect for a particular condition. Further study with larger samples is required to examine this subgroup, who may be phenotypically separate.

### What might underlie perceptual enhancement?

It remains unclear what underlies the enhancing effect of a sub-threshold. Iliopoulos et al. (2020) reported that a single subthreshold pre-pulse facilitates perception and is mediated by synchronization of mu-alpha rhythms in the somatosensory cortex whereby such synchronization of mu rhythms modulates perceptual processing. The mu rhythm is well recognized as an oscillatory mechanism to encode sensory tactile perception and shown to modulate with adaptation (Jones et al., 2007; Puts et al., 2019). Consequently, any modulation in the mu rhythms may act as a mediator for the effects of imperceptible stimuli as in pre-pulse stimulation of the present psychophysical task. TMS studies (in visual and auditory cortex) have shown that synchronization of the sensory cortex, by stimulating at the mu rhythm possibly towards an excitatory state, enhances perception (Song et al., 2011; Thut et al., 2011). Given that we are reproducing the enhancement of detection threshold by applying a pre-pulse, it is possible the pre-pulse induces changes in mu rhythm in neurotypicals but not in autistic children, which may be due to altered GABAergic signalling which has been shown to be associated both with the mu rhythm and with sensory perceptual differences (Jones et al., 2009; Puts et al., 2019).

Stimulus parameters also need consideration. In prior work, *repeated* administration of pre-stimulation was reported to shift the excitation-inhibition balance toward an inhibitory cortical state (reflected by perceptual impediment) accompanied by mu *de*synchronization (Iliopoulos et al., 2020). This is consistent with previous MEG work (Puts et al., 2019), and behavioural work showing that supra-threshold adaptation leads to impaired perception when the repeated stimulus is the same, but not when different, possibly due to engagement of inhibitory processes (Goble & Hollins, 1994; Mikkelsen et al., 2018; Puts et al., 2013; Tommerdahl et al., 2010) and these could explain the worsening of thresholds in the dynamic condition. These findings emphasise the importance of pre-stimulus properties, particularly single vs. repeated or dynamic administration of the prestimulus, as it seems this leads to different effects on detection thresholds and does not limit itself to our study but to physiological studies as well.

Neurotypical but not autistic children show improved detection thresholds in both paired-pulse conditions. Given the increasing amount of evidence of altered GABAergic function (Coghlan et al., 2012; J. Green & Garg, 2018; LeBlanc & Fagiolini, 2011; Rubenstein & Merzenich, 2003; Tavassoli et al., 2012), our findings suggests that that atypical GABAergic inhibition is influencing processing subthreshold pre-pulse stimuli in autism, which leads to ineffective sensory filtering. In neurotypicals it is possible that a pre-pulse primes the brain’s response to incoming stimulus to be assessed further, whereas in autism this mechanism is affected. While these effects are likely transient (on the order of milliseconds) and subject to specific stimulus parameters, they provide preliminary support that the inhibitory system is inefficient in autism. Tailoring stimulus parameters that are likely specific to individuals (we see some participants showing an improvement), might be consistent with individual optimized entrainment of cortical rhythms (Song et al., 2011; Thut et al., 2011).

The impact of ineffective sensory gating remains uncertain. While we could argue that poorer sensory gating impacts on sensory reactivity (e.g. a difficulty filtering our irrelevant information, or leading to sensory overload), we found no significant correlations between perceptual metrics and clinical features. The association between sensory filtering and sensory reactivity likely exists, but is not well captured by questionnaires, which are a subjective and historical assessment of concepts regarding sensory reactivity including perception and emotional responses. In larger samples, studies have shown a strong correlation between worse static detection threshold and increased hyper- and hyporeactivity (He et al., 2021; Tavassoli et al., 2016) but we were unable to reproduce this association, possibly due to the smaller sample. Further study is required in larger samples and with additional clinical metrics, such as adaptive function.

### Limitations and considerations for future work

Sensory differences in autism are multifaceted and the relationship between atypical perception, behavioural sensory differences and social communication remains poorly understood. The biology and behaviour of sensory differences is bi-directional and impacted by myriad of factors as symptoms severity, demographics, comorbidities, genetics and environment (J. Green & Garg, 2018; Lane, 2020; Mikkelsen et al., 2018; Piccardi et al., 2021). An in-depth account of these factors is beyond the scope of the study, but a direction for future research.

One limitation is that of the case-control design itself (Marquand et al., 2016). The case-control division introduces artificial symmetry and assumes that cases and controls are well-defined entities, not accounting for biological or symptom heterogeneity (Marquand et al., 2016). Future work should control for autistic traits and examine the impact of attentional difficulties, as well as mood on tactile performance.

A further limitation is that the sample in this study was predominantly of male participants which we have addressed in prior work for this sample (Asaridou et al., 2024). The few females were not excluded to retain participant numbers, however, future control and testing of gender effects would also increase the robustness of the findings.

### Implications and conclusions

Our findings suggest that atypical sensory gating in autism may be directly linked to perceptual differences, in this case, the detection of tactile stimuli, which we tested directly using psychophysical approaches. Future studies with larger samples can test this protocol and if pre-pulse stimulation, by modulating somatosensory processing, can be used to assess and modulate tactile hyper-hypo reactivities.

Our findings reflect the dynamic nature of sensory differences. We provide evidence that a single pre-pulse stimuli improves detection thresholds and as such, elicits opposing effects compared to previous studies showing effects of repeated pre-pulse stimuli which worsen detection thresholds. Further, in the current study and previous work (Puts et al., 2014), these opposing effects of single vs repeated/dynamic subthreshold stimuli are present in the neurotypicals, while the autistic groups are less affected and show no differences to baseline thresholds, reflecting differences in tactile sensory filtering. While these findings should be taken with caution given the limited sample size, they do provide important evidence towards understanding sensory differences as a dynamic system, rather than fixed trait of autism.

## Supplementary Material

1

## Figures and Tables

**Figure 1.**
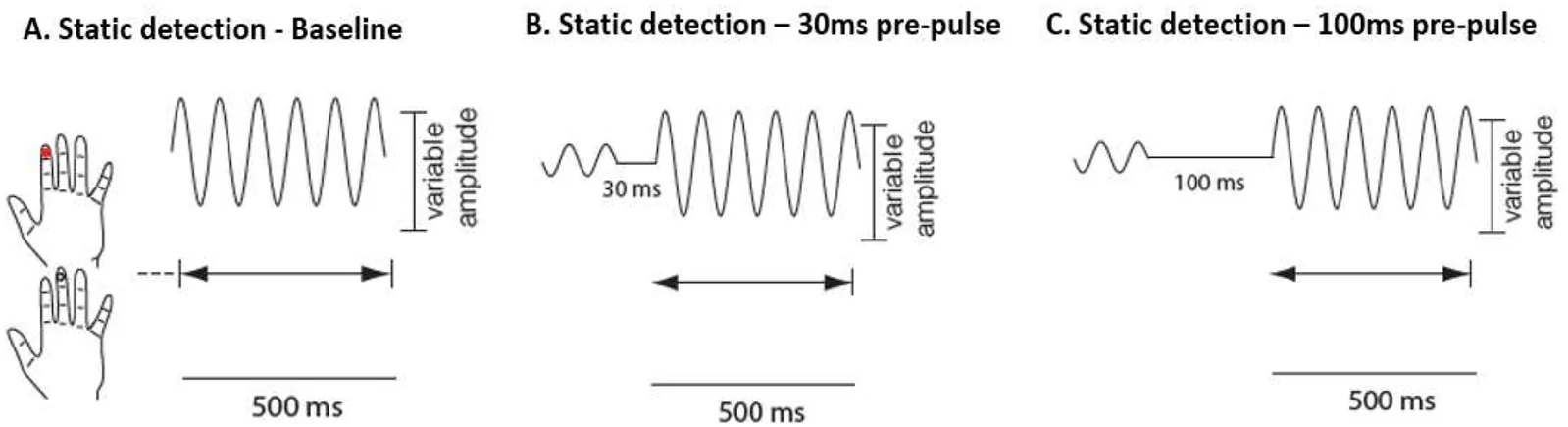
Visual schematic of the static detection protocol. **A** single tactile stimulus delivered to the child’s left index or middle finger. Upon accurate identification of the site of stimulation (left index or middle finger), the amplitude of the stimulus in the following trial would decrease, and this would make detection more difficult. If the participant failed to identify the correct site, then the amplitude of the stimulus in the next trial would increase, making detection of the stimulus easier. **B.** 30 ms prior to the primary stimulus as delivered at baseline, participants received a weaker “subthreshold” pre-pulse. **C.** 100 ms prior to the primary stimulus as delivered at baseline, participants received a weaker “subthreshold” pre-pulse.

**Figure 2.**
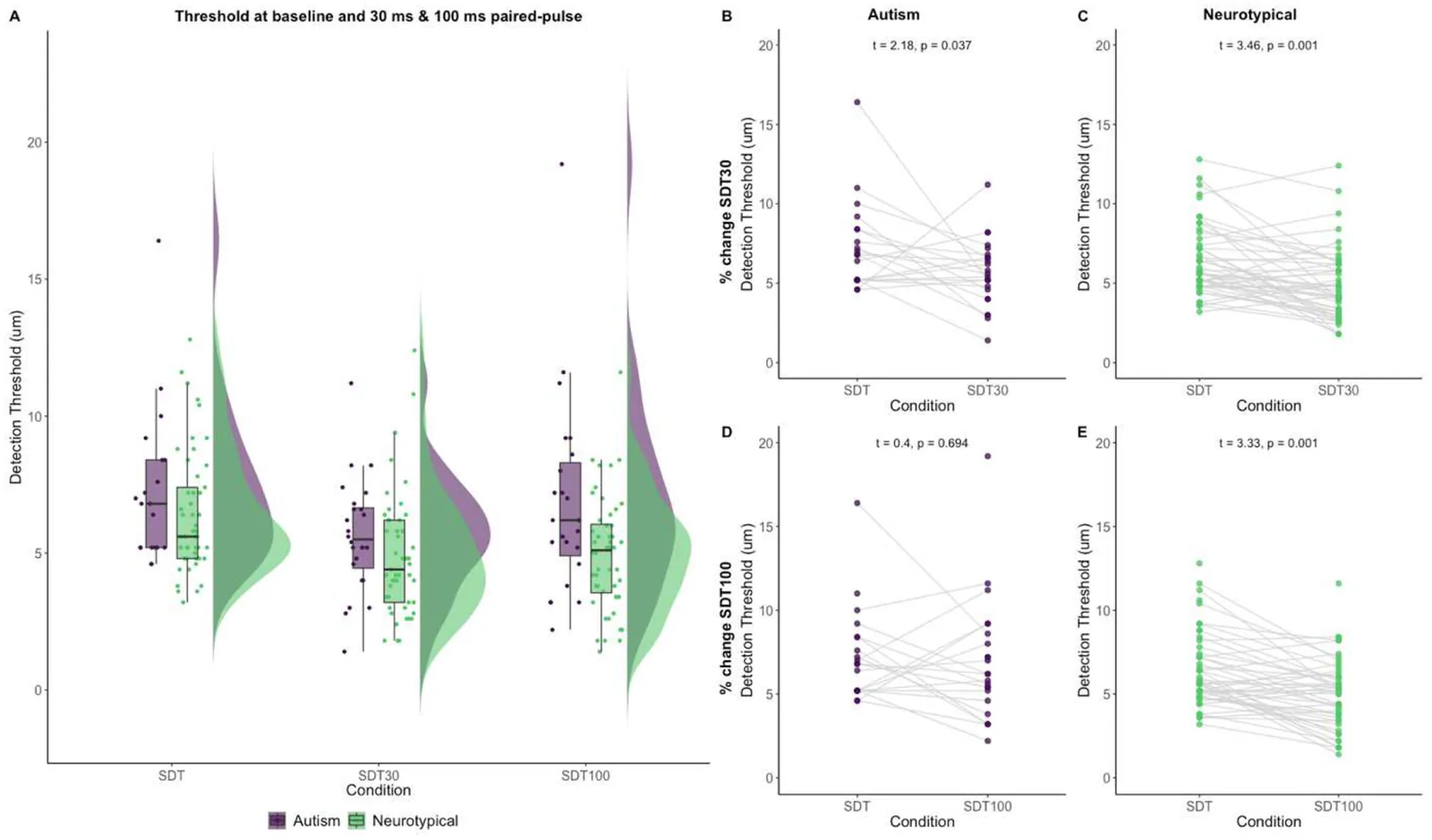
**A**. Individual data points with overlaid boxplots and density plots visualizing the group comparisons on detection threshold (μm) across the three conditions: baseline (SDT), 30ms pre-pulse (SDT30) and 100ms pre-pulse (SDT100). **B-C**. Individual line plots showing performance on detection threshold between baseline (SDT) and 30ms pre-pulse condition (SDT30) for autism (B) and neurotypical (C). **D-E**. Individual line plots showing performance on detection threshold between baseline (SDT) and 100ms pre-pulse condition (SDT100) for autism (D) and neurotypical (E).

**Figure 3.**
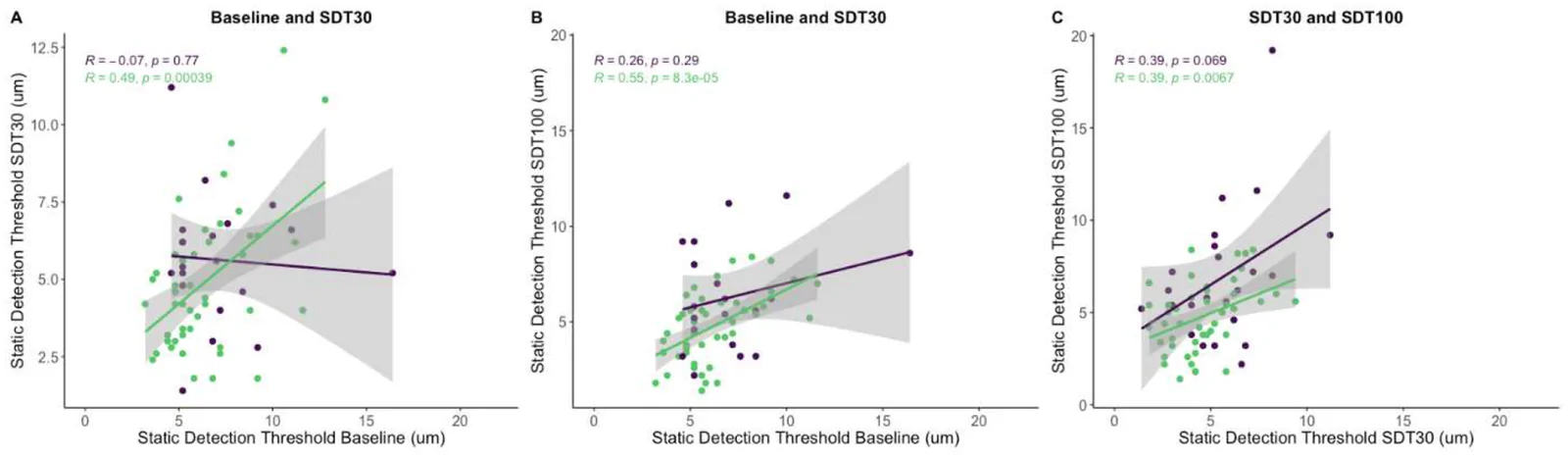
Correlations between static detection thresholds per diagnostic group **AB**. Baseline SDT and SDT30 and Baseline SDT and SDT100 correlate in neurotypical children but not in autistic children. **C**. Thresholds between both paired-pulse conditions correlate in neurotypical children, but not significantly in autistic children although they follow the same trend.

**Figure 4.**
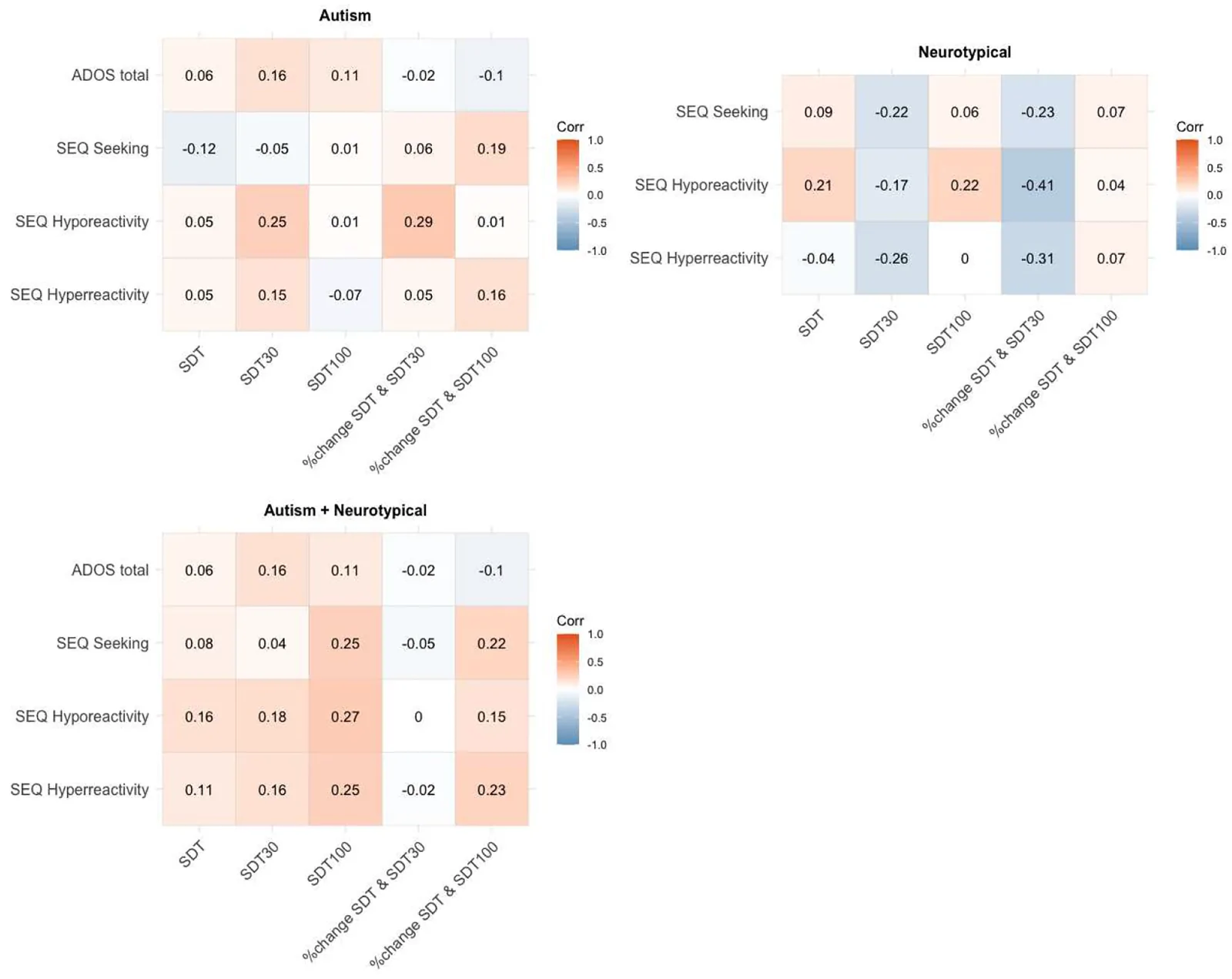
**A,B and C** Correlation matrix between SDT thresholds and % change scores with clinical metrics (SEQ and ADOS). No significant associations were found for the autism (A), neurotypical (B) groups or the entire cohort (C; note the association with ADOS total is only based on the autism group) (p > 0.05 for all).

**Table 1.** Demographics and descriptive statistics between autistic and neurotypical groups.

	Autism			Neurotypical			
	N	M	SD	N	M	SD	Sig.
Participants	24	-	-	51	-	-	-
Age	20	12.37	1.39	41	12.52	1.26	0.678
Gender (female)	3	-	-	11	-	-	-
Handedness (left)	3	-	-	4	-	-	-
***WISC- 5***							
Full scale IQ	24	105.54	15.96	51	112.90	11.17	0.050*
Fluid Reasoning Index	24	107.75	15.87	50	110.44	11.33	0.406
Visual spatial index	24	111.83	15.78	51	113.18	12.89	0.697
Processing speed index	24	97.42	15.78	51	105.50	12.82	0.021*
Verbal comprehension	24	103.91	15.79	51	111.51	11.60	0.043*
Working memory	24	104.12	15.61	51	110.59	12.45	0.057
***ADOS-2***							
Comparison severity score (CSS)	23	7.96	1.66	0	-	-	-
***SEQ 3.0***							
Total hyperreactivity	20	82.60	21.13	38	41.42	6.47	< 0.001*
Total hyporeactivity	20	31.80	9.66	38	21.66	3.76	0.029*
Total sensory seeking	20	69.25	19.86	38	42.95	10.31	< 0.001*

* values significant at α=0.05. For detection thresholds, see main analysis.

## Data Availability

All code will be made available on Open Science Framework and the BATD software is available as an R package (https://github.com/HeJasonL/BATD)

## Key points

- Pre-pulse inhibition improves detection thresholds in neurotypical children, but not in autistic children which indicates different perceptual capacity and sensory filtering mechanisms.
- Single pre-pulse stimuli elicit different effects compared to repeated pre-pulse stimuli protocols from previous studies which indicates that tactile perceptual responses are dynamic and stimulus dependent.
- Psychophysics protocols can test and modulate perceptual responses and as such, offer an objective and robust way of testing sensory perception.
