# Supplementary material for: Paired-pulse tactile stimulation enhances tactile perception in neurotypical but not autistic children
Source: Res Sq. 2026 Oct 6:rs.3.rs-11032804. Preprint. [Version 1] doi: 10.21203/rs.3.rs-11032804/v1 (PMC13692574; doi:10.21203/rs.3.rs-11032804/v1)
Supplement: Supplementary file 1 [file NIHPPRS11032804V1-supplement-1.pdf]

S1.1. Recruitment, Exclusion/ and Inclusion Criteria

Participants were recruited through advertisements in schools, advocacy organizations and medical institutions. Preliminary screening was done by telephone interviews with the parents

Inclusion Criteria

Neurotypical: free of psychiatric disorders as measured by DICA -IV (Reich, 2000) or/and the Kiddie Schedule for Affective Disorders and Schizophrenia (Kaufman et al., 1997).

Autism: met DSM-5 and/or DSM IV criteria. Diagnosis based on clinical judgement and confirmed with ADOS-2 and/or ADI-R.

Exclusion Criteria

- Intellectual disability
- Seizures
- Brain injury
- Other neurological disorders (e.g., Tourette's)
- Children taking psychotropic medication other than stimulants. Children who were on stimulant medication, stopped talking the medication on the day prior and the day of testing.
- IQ scores below 80. In cases of a discrepancy greater than 12 points, the Fluid Reasoning Index and Visual Spatial Index of WISC-5 was required to be over 80 and the lower of the two was required to be over 65.

S2. Participants with "worse" static detection performance

Table S2. Characteristics of participants with "worse" static detection performance in pre-pulse conditions. No significant group differences on SEQ and ADOS-2 scores.

**Characteristics of participants with "worse" static detection performance in pre-pulse conditions**							
							**N**
Total							28
Number of participants with worse performance in only one pre-pulse condition							19
30ms condition							8
100ms condition							11
Number of participants with worse performance in both pre-pulse conditions							9
Autistic							10
Neurotypical							18
	**Improvement**			**Worse performance**			
	N	M	SD	N	M	SD	Sig.
SEQ – hyperreactivity	37	1.76	0.77	21	1.84	0.77	0.722
SEQ – hyporeactivity	37	1.41	0.47	21	1.67	1.33	0.272
SEQ – sensory seeking	37	1.68	0.61	21	1.67	0.65	0.937
ADOS -2 total score	14	15.85	4.43	9	13.22	3.11	0.137

S3.1 Figure including dynamic detection threshold

Figure S3.1 Figure also showing heightened dynamic detection threshold (DDT) from a separate cohort, showing increased dynamic detection threshold in neurotypical children compared to SDT and single pre-pulse conditions, but not in autistic children.
